# Supplementary material for: Whole body physiology model to simulate respiratory depression of fentanyl and associated naloxone reversal
Source: Commun Med (Lond). 2024 Jun 12;4:114. doi: 10.1038/s43856-024-00536-5 (PMC11169242; doi:10.1038/s43856-024-00536-5)
Supplement: Supplementary file 2 — Supplemental Information [file 43856_2024_536_MOESM2_ESM.docx]

Supplemental Information

Austin Baird^1*^, Steven A. White^2^, Rishi Das^2^, Nathan Tatum^2^, Erika K Bisgaard^3^

^1^University of Washington Department of Surgery, Division of Healthcare Simulation Sciences, Seattle, Washington, USA

^2^Applied Research Associated Southeast Division, Raleigh, North Carolina, USA

^3^University of Washington Department of Surgery, Division of Trauma, Burn, and Critical Care Surgery, Seattle, Washington, USA

# Supplemental Note 1 – write up

Transport (Cardiovascular, Respiration and substance)

The solver performs numerical integration through linearization (first order approximations) by assuming a direct current (DC) solution for the given time step, Supplemental Figure 1. The Modified Nodal Analysis (MNA) approach is used to determine the state of every node and path within the circuit. The steps for solving a circuit in each time step are:

1. Perform numerical integration by using linearization (first order approximations) through MNA, using the matrix algebra equation Ax=b.
2. Use Kirkoff’s Current Laws (KCL), figure 1, (sum of the currents is zero at each node) to populate the A matrix and b vector. Figure 2 shows the equations used for determining flows, where flows (F) are equivalent to currents, and pressures (P) are equivalent to voltages, Supplemental Figure 2.
3. Leverage the Eigen templated library LU decomposition linear solver to solve for unknown variables (i.e., voltages and currents) given variable pressures generated through the driver functions. This decomposition represents an LU decomposition of the constructed matrix with complete pivoting.
4. Calculate remaining unknown currents using the Trapezoidal Rule, where applicable. For nonlinear elements from time a to b.
5. Calculate diode currents using assumed open or closed switch states (cannot be solved directly). Iterate Steps 1–3 until a satisfactory solution is attained.
6. Calculate the change in charge (Q) across capacitors based on the capacitance (C) and voltage (V) change and increment the total charge. The charge is conserved on the source and target nodes by incrementing/decrementing this amount. Selecting which node gains charge and which loses is done by the direction of the current in the path.

$$Q(t)=CV(t)$$

1. Invalidate the current on any path the user has specified to ignore. Note that this is to prevent unwanted transport to the reference node (i.e., ground).

Respiratory Driver

The BioGears Respiratory System interacts with other systems in the engine to receive feedback and adjust spontaneous breathing for homeostasis. To accurately model the respiratory response under various physiological and pathological conditions, a robust Respiratory Model that responds to mechanical stresses and chemical stimuli is required. To this end, the BioGears Respiratory System employs a time-dependent pressure source based on a chemical feedback mechanism that mimics the respiratory response to blood gas levels as sensed by the central and peripheral chemoreceptors. The pressure source represents the muscle pressure source signal and serves as an input power source to drive the inspiration and expiration phases of the breathing cycle.

During inhalation, the driver pressure source is set to a negative value. The end of the exhalation cycle represents the initial conditions of free breathing, where the alveolar pressure equals the atmospheric pressure and no air flows into the lungs. When the inspiratory muscles are not contracting, the mechanical interaction between the lungs and the chest wall creates a sub-atmospheric intrapleural pressure. The value of the driver pressure in the model is selected to meet the unstressed condition at the pleural node.

For a realistic muscle pressure source signal, the BioGears Respiratory System adopted the mathematical model proposed by which is based on clinical data. Accordingly, the time series of the respiratory muscle pressure, *Pmus*, is given by,

$$P_{mus}=\left\{\begin{matrix} -\frac{P_{\max}}{IE}t^{2}+\frac{P_{\max}T}{IE}t,0<t\leq I \\ \frac{P_{\max}}{1-\exp\left(\frac{E}{\tau}\right)}\left(\exp\left(\frac{t-I}{\tau}\right)-\exp\left(\frac{-E}{\tau}\right)\right),I<t\leq T \end{matrix}\right.$$

*Equation 6.*

Where I, E, and T are the inspiratory, expiratory, and total respiration times, respectively. The value τ is a time constant for the expiration period and is estimated as E / 5. The total breathing cycle time T is obtained from the inverse of the respiration rate determined by the [chemoreceptor model](https://www.biogearsengine.com/documentation/_nervous_methodology.html#nervous-features-chemoreceptors). I and E are calculated using the inspiratory: expiratory ratio from the previous time step, which is modified by irregular physiology like asthma and COPD. The chemoreceptor model also updates the driver amplitude, Pmax. The baseline value of Pmax for each virtual patient is determined during engine initialization by modifying the amplitude at the requested patient respiration rate until a stable tidal volume is obtained.

Substance Transport

Given the overall goals of BioGears, we set out to create a generic and reusable substance transporter. Some high-level requirements include:

- Generic - All systems should be able to use the same basic transporter engine. This allows rapid development and makes engine outputs much easier to validate and verify.
- Computational Speed - BioGears is required to maintain a transient full-body solution faster than real time on typical personal computers.
- Modular - Using the same basis for design and construction will aid in keeping the system decoupled.
- Extensible - We must take future growth into consideration and allow users and developers the proper tools and building blocks on which to add new functionality.
- Dynamic - Feedback mechanisms are required for each system. It is beneficial to be able to dynamically change, add, or remove compartments and links.
- Conservation - We must uphold sound scientific principles and conserve mass.
- Common Data Model - The entire solution must reside within and effectively use the [Common Data Model](https://www.biogearsengine.com/documentation/_c_d_m.html).
- Fluid types - BioGears will include liquid and gas systems. It is beneficial to use the same solver for both types.
- Bifurcations - Each compartment can have an unlimited number of links providing flow/substances both in (up stream) and out (down stream).
- Large flows - The transporter must be able to handle any size flows, including instances where significantly more volume is moved than exists in a given compartment for a given time-step.

The Transporter is implemented generically for both liquid and gas systems using the same high-level definitions for substance properties, which are:

- Extensive property: additive for independent, non-interacting subsystems - proportional to the amount of material in the system
  - Property changes with amount change
  - Pouring some out will change the value
  - Examples: Mass, Volume, Length, Amount
- Intensive property: a bulk property, meaning that it is a physical property of a system that does not depend on the system size or the amount of material in the system
  - Property doesn't change with amount change
  - Defines and identifies substances
  - Pouring some out will not change the value
  - Examples: Concentration, VolumeFraction, and Temperature

The Transporter assumes that fluid movement (i.e. convection) has already taken place - generally calculated and updated inside the system by the circuit solver. Once the convective fluid movement properties of compartment volume and link flow are updated for the current time-step being analyzed, the extensive and intensive substance values at each compartment can be determined by using the previous time-step state. Supplemental Table 2 shows the parameters needed to calculate the advective transport by bulk flow.

The instantaneous substance quantity values can be determined in each compartment of a graph by doing a mass balance calculation where *m* is the mass on the current compartment and both *min* and *m_out_* are provided by links to any number of other compartments:

$$\sum m_{in}-\sum m_{out}=\Delta m$$

Using table 1 we can break this equation down into its components:

$$\sum I_{SC}f_{IL}t-\sum I_{C}f_{OL}t=I_{C}V_{C}-E_{o,C}$$

We can then rearrange this equation to obtain:

$$I_{C}V_{C}-\sum I_{SC}f_{IL}t+\sum I_{C}f_{OL}t=E_{o,C}$$

By combining these equations for all compartments in the graph, a set of linear equations can be written in the form of Ax=b to solve for the new intensive properties. Here, A is a matrix of constants that is invertible, x is a vector of intensive properties and b is the know previous time step properties.

The algorithm, implemented in C++ in the BioGears engine, used by the transporter to solve a graph using 1.4 for each time-step are:

1. Loop over compartments to populate the A matrix (one row per compartment) - this is the same for all substances.
   1. Handle infinite volume (often the environment) by setting intensive property constant.
   2. Handle no volume by using an approximate zero (1e-20) value to prevent a singular matrix.
   3. Handle source link flows (out of the compartment)
   4. Handle target link flows (into the compartment)
2. Loop over all substances
   1. Populate b vector with previous time-step masses.
   2. Solve for x vector intensive properties.
   3. Parse intensive properties and calculate new extensive properties.

Each individual system uses the Calculate Substance Transport function to generically complete transport during the Process step, which is an intermediate compute step between timesteps and after the generic fluid calculations.

In addition to transport, BioGears implements a generic substance definition to aid in extending the engine for additional substances (additional opiates may be implemented using these definitions), Supplementary Figure 7.

Cardiovascular Function

The cardiovascular model is regulated by the baroreceptors, which work to regulate elastance during a cardiac cycle. Although not affected by the opioid directly the cardiac output is seen to increase for large opiate use scenarios, Supplemental Figure 3. This corresponds to the decrease in blood pressure (seen by the decrease in overall systemic resistance) and the slight reduction in heart rate during the early stages of the fentanyl progression. Cardiac output in the physiology engine is computed to be the stroke volume multiplied by heart rate. Even though the heart rate decreases slightly for high levels of opioid use, the stroke volume increases due to lower blood pressure. Stroke volume and most systemic cardiovascular effects are unchanged for opiate doses below the 1 mg threshold. In addition, as oxygen is depleted due to the respiratory depression, the nervous system adjusts the resistance into the myocardium increase fluid flow into the heart muscle tissue to maintain oxygenation.

Fentanyl pharmacodynamics provide some depression to the main drivers of the circulation, namely heart rate and blood pressure. Although small, these changes can be seen for opioid doses over 1 mg, Supplementary Figure 4. As the opiate is cleared in the body and the respiratory depression is reversed with naloxone, the nervous system can compensate for the lack of oxygen in the blood by spiking the heart rate. We note that this increase is moderate and is an artifact of the injury that the patient has just sustained during the overdose episode. Relaxation of the heart and the vascular resistance is seen as the patient fully recovers at the end of the episode.

Additional cardiovascular markers follow this trend. An increase in output, followed by a relaxation period as respiratory depression is normalized due to the Naloxone treatment. Cardiac output and vascular resistance are two such metrics. The two measurements follow almost mirror image trajectories throughout the treatment period. Cardiac output sees a nearly linear response due to stroke volume and systemic vascular resistance changes. Even though the heart is not beating as frequently, the volume of fluid is increasing due to the resistance changes downstream. Oscillations seen are due to the forcing on the system through the nervous baroreceptor model. Larger oscillations are seen in the higher Fentanyl doses because the work on the system is increasing due to the reduction in oxygen transport in the system.

Supplementary Table 1 – pharmacodynamic parameters available in the biogears engine, coupled with their definition.

| Effect Name | Description |
| --- | --- |
| Effect Site Rate Constant | Describes the speed of equilibrium between plasma concentration and site of action of a drug |
| Emax Shape Parameter | Adjusts the sigmoidal response in the deltaE functional relationship |
| Antibacterial Effect | The enhancement in the bacterial death rate caused by an antibiotic |
| Bronchodialation | Dilation of the trachea resistance pathway in the respiratory circuit |
| Central Nervous Modifier | Drug depressant effect on receptors in the central nervous system directing chemoreceptor action |
| Diastolic Pressure | Change in diastolic pressure |
| Heart Rate | Change in heart rate |
| Hemorrhage Flow | Change in the hemorrhage bleeding rate (flow to ground from the vascular structure) |
| Neuromuscular Block | Effect to cause paralysis |
| Pupil Size Modifier | Effect on pupil size |
| Pupil Reactivity Modifier | Effect on reactivity to light |
| Sedation | Effect to cause sedation (during ventilation) |
| Systolic Pressure | Change in systolic pressure |
| Tidal Volume | Adjustment to the possible tidal volume the patient can achieve |
| Tubular permeability | Localized effect on tubular permeability in the renal system |

Supplementary Table 2 - Clearance and physiochemical properties of naloxone. Some parameters have small deviations from reported literature but are generally within the error deviation reported. Changes are due to matching concentration profiles of naloxone in the blood.

| Systemic Clearance Values | | | Physiochemical properties | | |
| --- | --- | --- | --- | --- | --- |
| Name | **Value (mL/min kg)** | **Reference** | **Name** | **Value** | **Reference** |
| Fraction Unbound In Plasma | 0.62 | (Blanchard et al., 2006) | **Acid Dissociation Constant** | 7.94 | (PubChem, n.d.) |
| Intrinsic Clearance | 400 | (Mistry and Houston, 1987) | **Binding Protein** | Albumin | (PubChem, n.d.) |
| Renal Clearance | 0.5 | Computed | **Blood Plasma Ratio** | 1.08 | (Mistry and Houston, 1987) |
| Systemic Clearance | 27.5 | (Ziesenitz et al., 2015a) | **Ionic State** | Weak Base | (PubChem, n.d.) |
|  |  |  | **LogP** | 1.92 | (Wermeling, 2013) |

Supplementary Table 3 - Kinetic rate parameters used for the nasal administration model

| Symbol | Value | Symbol | Value |
| --- | --- | --- | --- |
| k_1_ | 0.00001736 | **k_9_** | 1000000 |
| k_2_ | 1000000 | **k_10_** | 0.000000027 |
| k_3_ | 0.00001736 | **k_11_** | 0.00001 |
| k_4_ | 0.000173 | **k_12_** | 0.00001 |
| k_5_ | 0.0011575 | **k_13_** | 0.00001 |
| k_6_ | 1000000 | **k_14_** | 0.0000278 |
| k_7_ | 0.0011575 | **k_15_** | 0.0000278 |
| k_8_ | 0.0000260 |  |  |

Supplemental Table 4 - p-values reported for average naloxone dose required between the given fentanyl ranges reported. Values calculated via the medcalc online utility.

| Fentanyl Range Comparison | p-value |
| --- | --- |
| 0.2-0.7 v 0.8-1.3 | 0.0006 |
| 0.8-1.3 v 1.4-1.9 | 0.0018 |

| 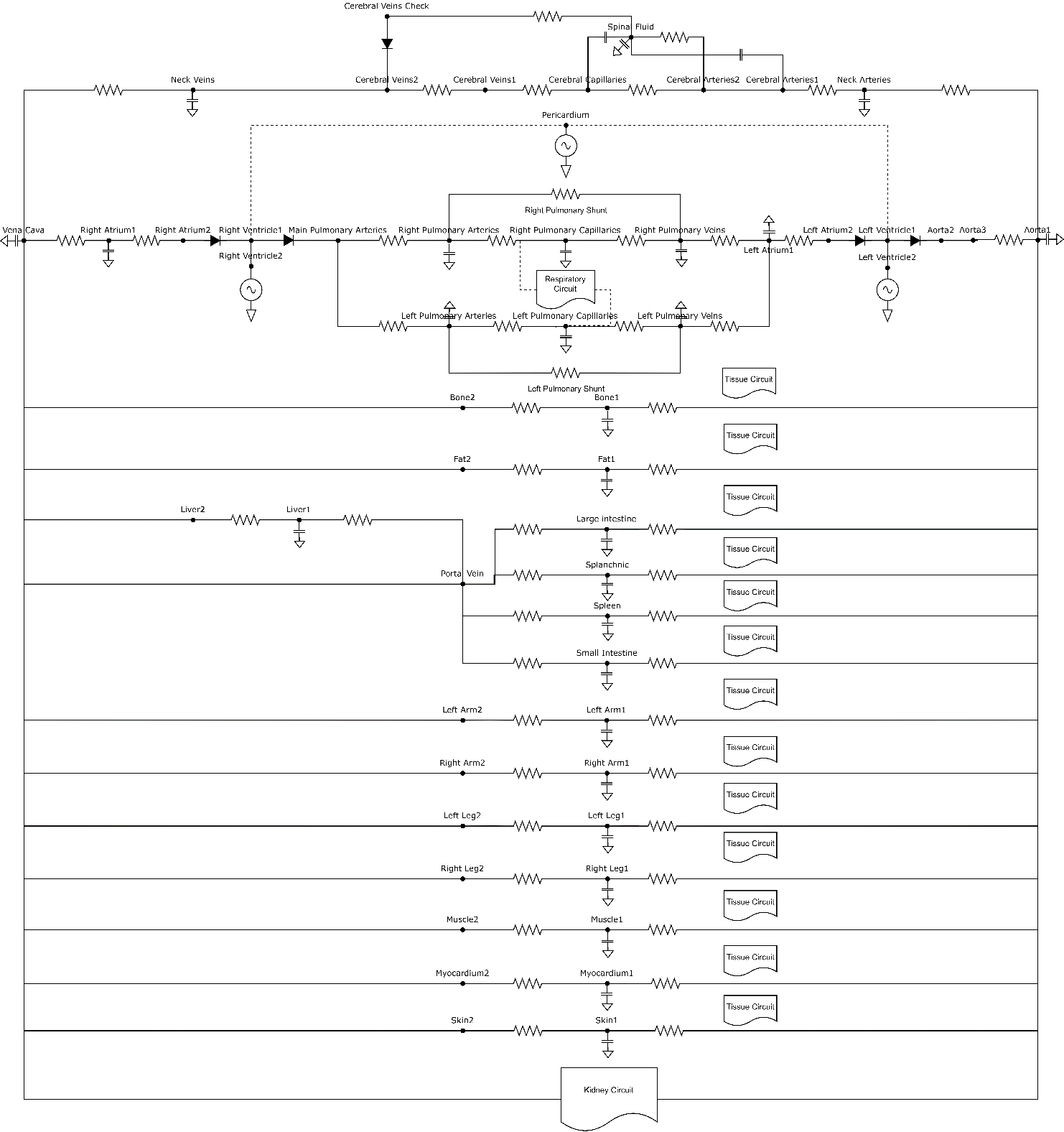 |
| --- |
| Supplementary Figure 1 – Circuit diagram of the BioGears circulatory system. The additional boxes denote broken out circuit structure for each of the noted biological system, the renal, tissue, and respiratory (gas) systems have their own separate circuits that connect to the vasculature through various junctions not shown here. |

| 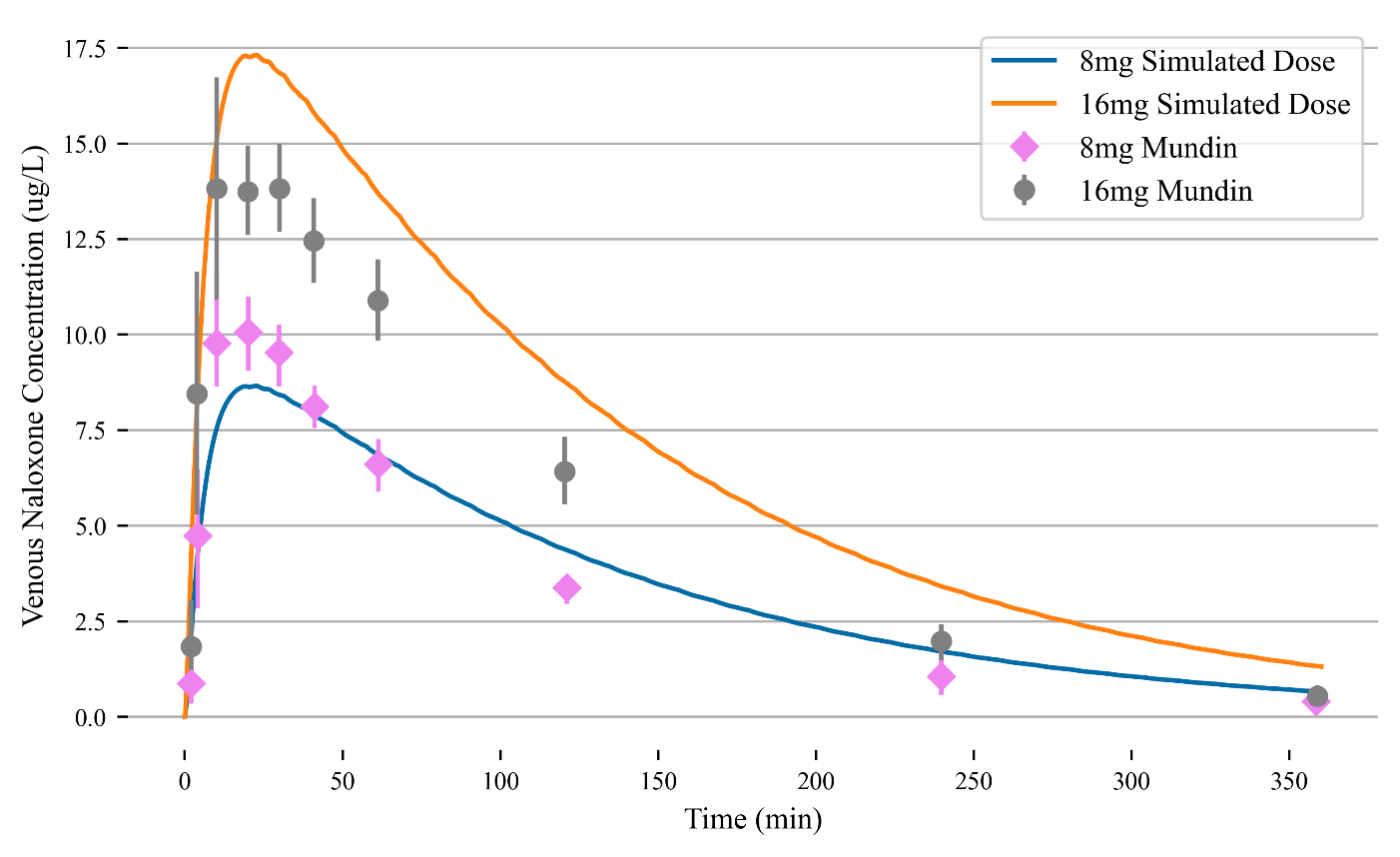 |
| --- |
| Supplementary Figure 2– 6-hour simulation of naloxone for 16 mg (orange) and 8 mg (blue) metered nasal spray doses. 16 mg simulated data clearly overshoots the peak profile of the experimental data. Both simulations match the early onset growth and long-term dynamics well. |

| 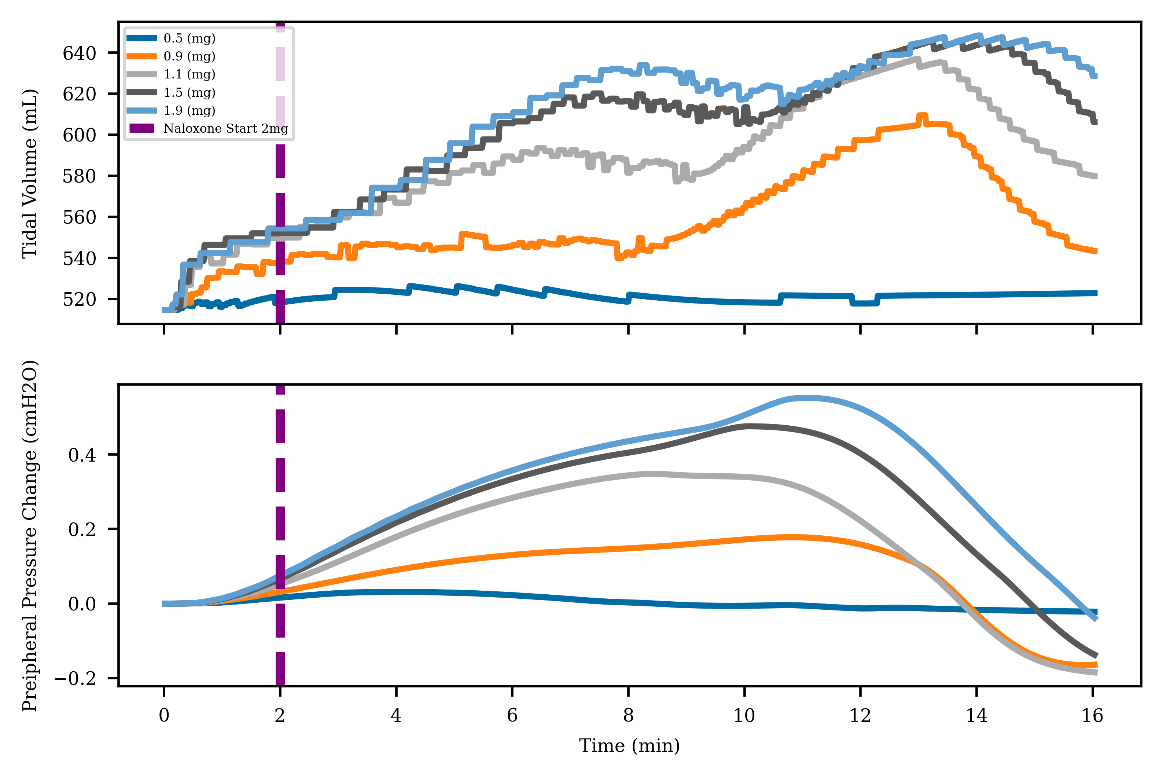 |
| --- |
| Supplementary Figure 3 – Peripheral pressure changes react to the reduction in oxygen and correspondingly provide changes to the respiratory driver pressure, leading to an increase in tidal volume . |

| 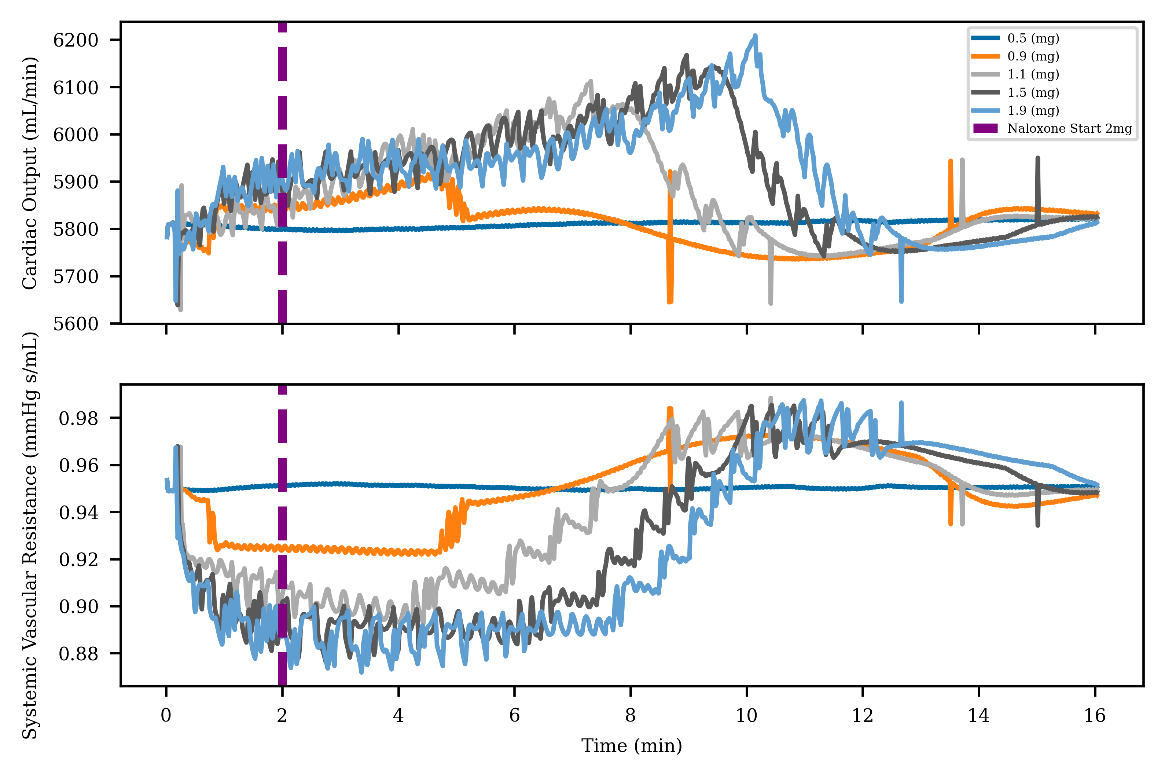 |
| --- |
| Supplementary Figure 4 – Cardiac output and systemic vascular resistance seen for varying levels of fentanyl administration. Rising cardiac output tries to compensate for the reduction in oxygen content in the blood. |

| 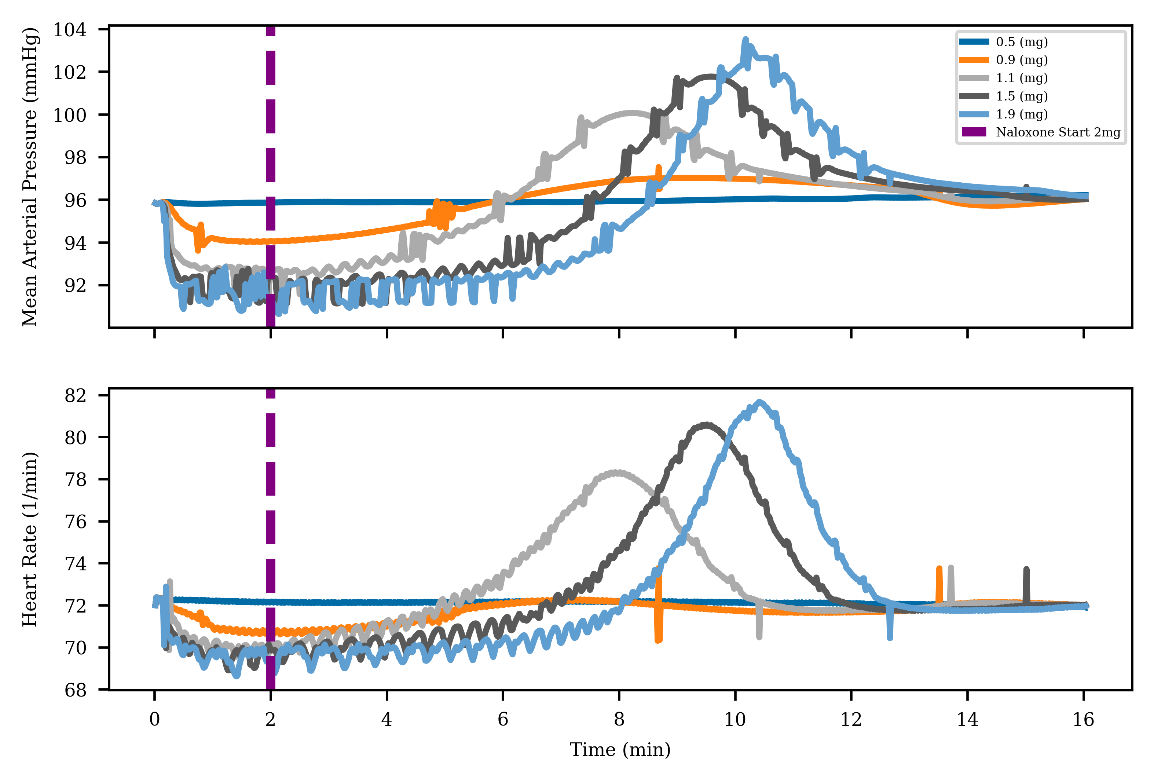 |
| --- |
| Supplementary Figure 5 – Mean atrial pressure and heart rate for varying levels of opioid administration in the patient. Initial depression is due to the opioid with a recovery period following clearance and reversal. |

| 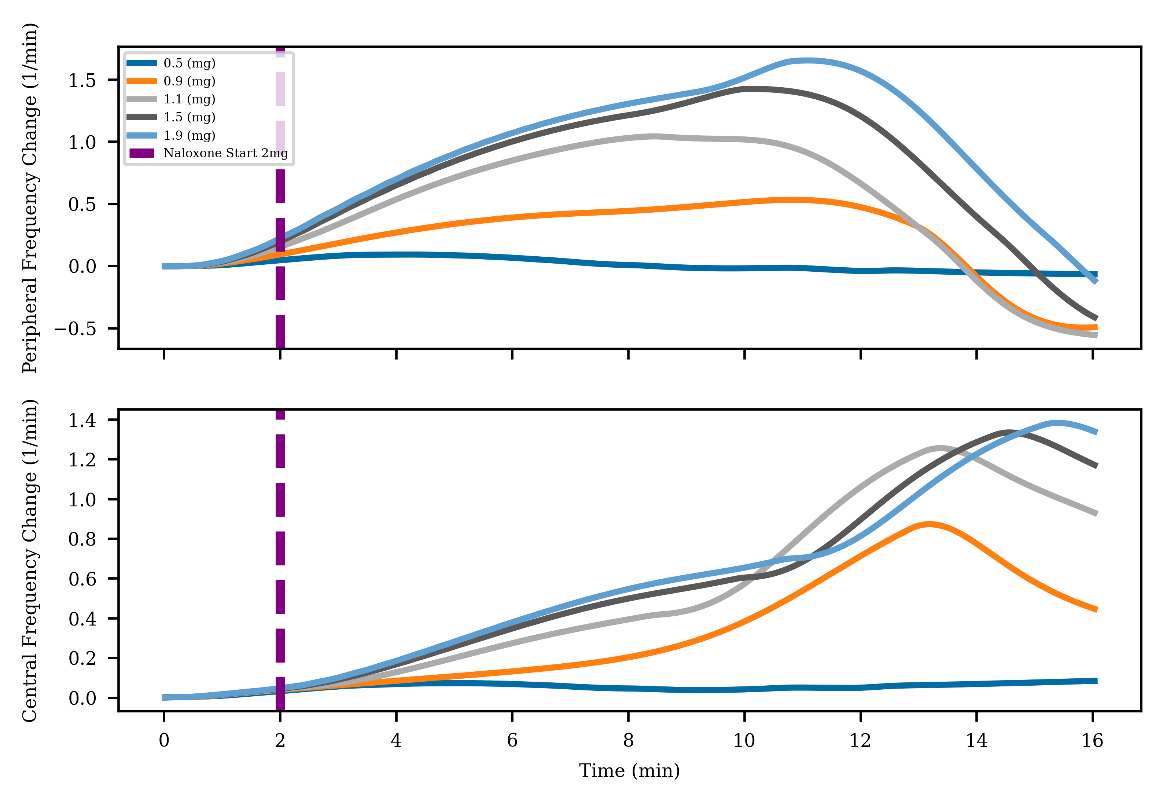 |
| --- |
| Supplementary Figure 6 – Central frequency function is reduced during the initial onset of the opioid. Because the opioid only targets the central nervous system, the peripheral frequency responds to the overdose event. |

| 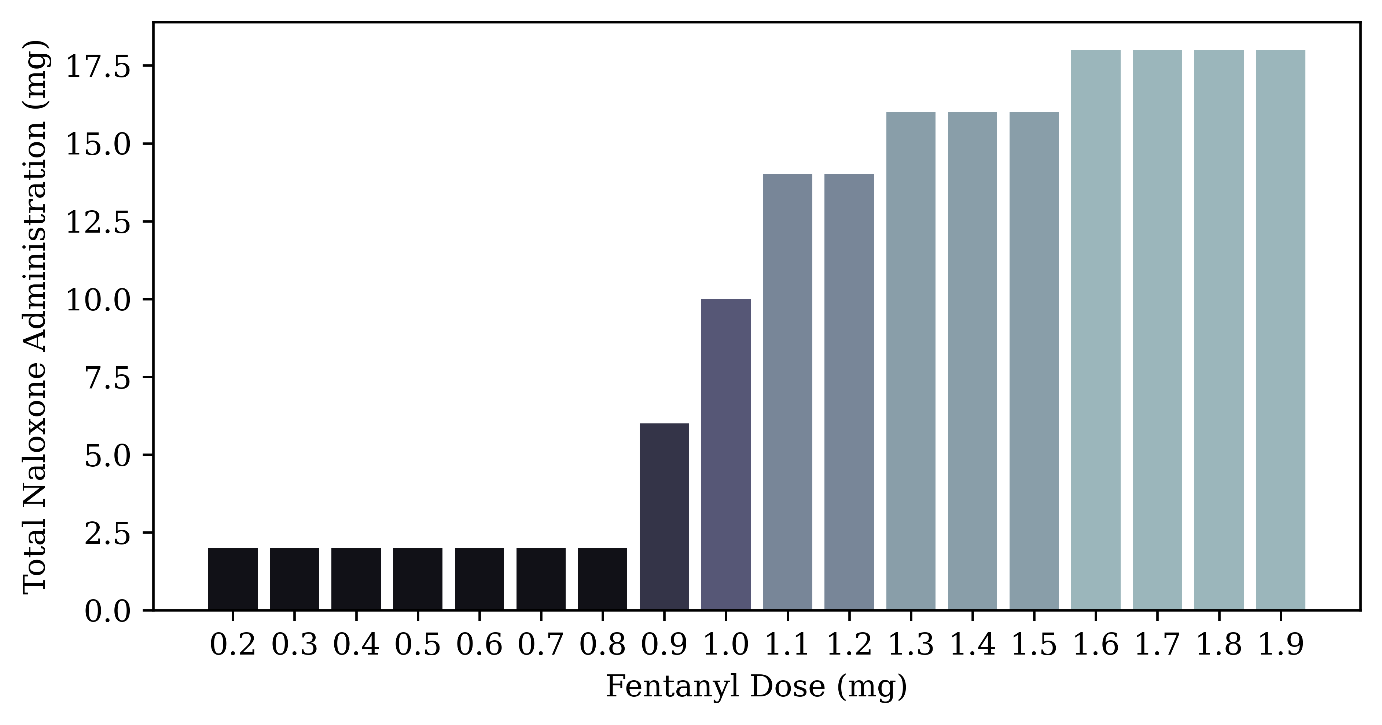 |
| --- |
| Supplementary Figure 7 – Total rescue dose administered for each fentanyl dose. A nonlinear response is seen as we transition from 0.8 to 1.1 mg amounts. Bars are colored as a function of total naloxone dose. |


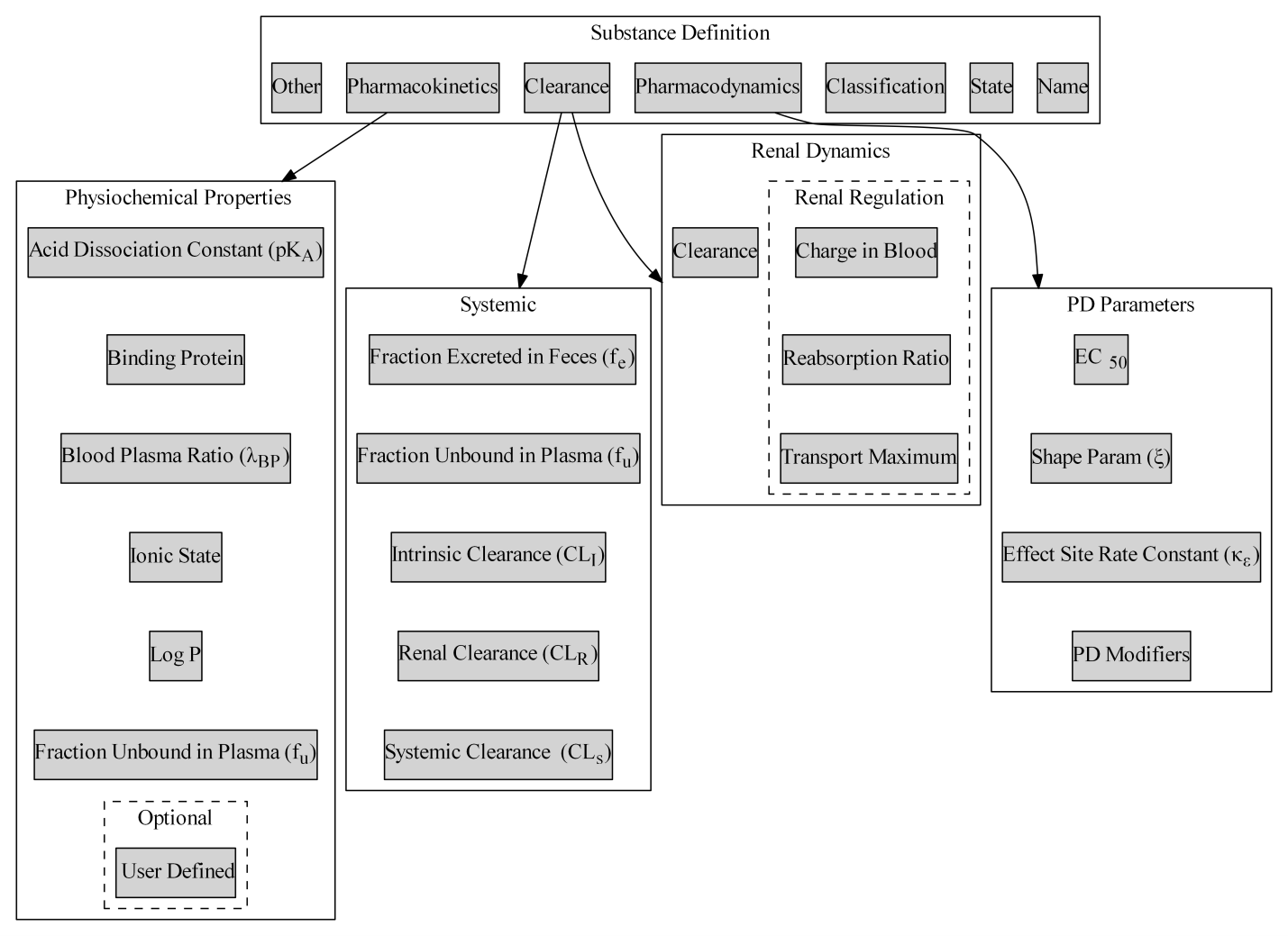


Supplementary Figure 8 – Generalized substance definition standard that is used to create substances in the BioGears engine. The definition defines how a substance is handled amongst various models implemented in the engine.
